# (1‐aryloxy‐2‐hydroxypropyl)‐phenylpiperazine derivatives suppress *Candida albicans* virulence by interfering with morphological transition

**DOI:** 10.1111/1751-7915.13307

**Published:** 2018-09-17

**Authors:** Shuo Zhao, Jun‐Jun Huang, Xiuyun Sun, Xiaorong Huang, Shuna Fu, Liang Yang, Xue‐Wei Liu, Fei He, Yinyue Deng

**Affiliations:** ^1^ State Key Laboratory for Conservation and Utilization of Subtropical Agro‐Bioresources South China Agricultural University Guangzhou 510642 China; ^2^ Guangdong Innovative Research Team of Sociomicrobiology College of Agriculture South China Agricultural University Guangzhou 510642 China; ^3^ Integrative Microbiology Research Centre South China Agricultural University Guangzhou 510642 China; ^4^ Pharmaceutical Research Center School of Pharmacology Guangzhou Medical University Guangzhou 510182 China; ^5^ Singapore Centre for Environmental Life Sciences Engineering (SCELSE) Nanyang Technological University Singapore 637551 Singapore; ^6^ Division of Chemistry and Biological Chemistry School of Physical and Mathematical Sciences Nanyang Technological University 21 Nanyang Link Singapore 637371 Singapore

## Abstract

Clinical treatment of *Candida albicans* infections has become more difficult due to the limited development of antifungal agents and the rapid emergence of drug resistance. In this study, we demonstrate the synthesis of a series of piperazine derivatives and the evaluation of their inhibitory activity against *C. albicans* virulence. Thirty‐four (1‐aryloxy‐2‐hydroxypropyl)‐phenylpiperazine derivatives, including 25 new compounds, were synthesized and assessed for their efficacy against the physiology and pathogenesis of *C. albicans*. Several compounds strongly inhibited the morphological transition and virulence of *C. albicans* cells, although they did not influence the growth rate of the fungal pathogen. A leading novel compound, (1‐(4‐ethoxyphenyl)‐4‐(1‐biphenylol‐2‐hydroxypropyl)‐piperazine), significantly attenuated *C. albicans* virulence by interfering with the process of hyphal development, but it showed no cytotoxicity against human cells at a micromolar level. These findings suggest that (1‐aryloxy‐2‐hydroxypropyl)‐phenylpiperazine derivatives could potentially be developed as novel therapeutic agents for the clinical treatment of *C. albicans* infections by interfering with morphological transition and virulence.

## Introduction

*Candida albicans* has become the most frequently encountered fungal pathogen, causing mild mucosal infections and superficial but often persistent oral or vaginal candidiasis in humans (Poulain, 2015). It can also induce life‐threatening systemic infections known as candidemia in immunocompromised patients (Miller *et al*., 2001; Pappas *et al*., 2003). *C. albicans* has a distinguishing feature, the yeast‐to‐hyphae dimorphism, which is the most important virulence factor that enables *C. albicans* to infect humans (Madhani and Fink, 1998; Brown and Gow, 1999; Staib *et al*., 2000). During the initial stage of infection, *C. albicans* cells exhibit a planktonic yeast morphology that is avirulent, and a subsequent transition from yeast to hyphae leads to tissue invasion in patients (Sudbery *et al*., 2004; Saville *et al*., 2003; Lo *et al*., 1997; Finkel and Mitchell, 2011). This ability to switch between yeast and hyphae is indispensable for the pathogenesis of *C. albicans* (Noble *et al*., 2017).

The clinical treatment of candidiasis caused by *C. albicans* relies on limited drugs, which are usually composed of three major classes: polyenes, azoles and echinocandins (Odds *et al*., 2003; Pierce *et al*., 2013). However, problems such as selectivity, toxicity and the development of resistance have led to an emergent need for new strategies and novel drugs to efficiently treat *C. albicans* infections (Chandra *et al*., 2001; Ramage *et al*., 2002; Tobudic *et al*., 2010). Several categories of natural products and synthetic chemicals have been screened and evaluated for their efficacy against *C. albicans* adhesion and morphological transition in recent years. Carvone and perillaldehyde were found to inhibit the formation of *C. albicans* filamentous structures (McGeady *et al*., 2002). Quorum sensing molecules such as farnesoic acid and *cis*‐2‐dodecenoic acid (BDSF) inhibit *C. albicans* hyphae formation and appear to play a key role in regulating the *C. albicans* morphological transition (Oh *et al*., 2001; Kim *et al*., 2002; Boon *et al*., 2008; Deng *et al*., 2010). The recently identified small molecule filastatin exhibits excellent inhibitory activity against the cell adhesion, morphogenesis and pathogenesis of *C. albicans* (Fazly *et al*., 2013).

Piperazine derivatives were reported to exhibit extensive pharmacological activities, such as antifungal, antioxidative and antitumor activities and the inhibition of cardiovascular disease (Huang *et al*., 2015, 2016; Gettys *et al*., 2017). These previous findings motivated us for the further exploration of the pharmacological activity of piperazine derived compounds. In this study, we reported the chemical synthesis of 34 piperazine derivatives, including some novel compounds, for the first time and investigated their ability to inhibit *C. albicans* virulence. Some derivatives showed excellent efficacy in preventing yeast‐to‐hyphae transition, biofilm formation and virulence but did not interfere with the growth rate of *C. albicans* cells. Intriguingly, these compounds were nontoxic to human cells, even at a high concentration. Together, our methods focus on evaluating multiple pathogenesis‐related functions that do not involve the death of fungal pathogen cells to promote the development of novel antifungal therapeutics against *C. albicans*.

## Results

### Chemical synthesis

**Figure 1:**
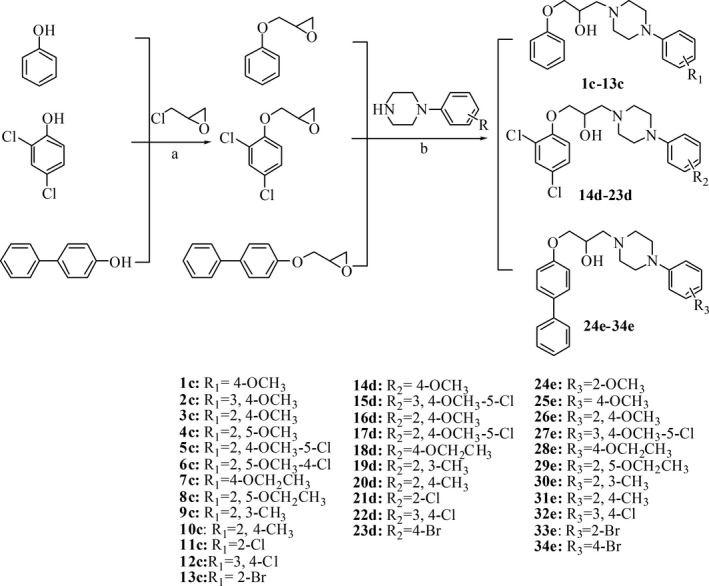
Chemical synthesis of piperazine compounds **1c‐34e**. A. NaOH, 0–60°C. B. 2‐propanol, reflux.

Piperazine derivatives **1c‐34e** were synthesized following the methods outlined in Fig. 1 (Pollard and Christie, 1958; Huang *et al*., 2014). Phenol, 2,4‐dichlorophenol and 4‐hydroxybiphenyl were the initial precursors, which were then converted into epoxides through nucleophilic substitution with epichlorohydrin, followed by substitution with different piperazines in 2‐propanol to yield the target compounds **1c‐34e** (Fig. 1, Table S1). The derivatives were characterized based on ESI‐MS, HRESI‐MS and ^1^H NMR spectral data, the results of which were completely consistent with their depicted structures (Fig. 1, Table S1).

### Piperazine derivatives inhibit hyphae formation in *C. albicans*

**Figure 2:**
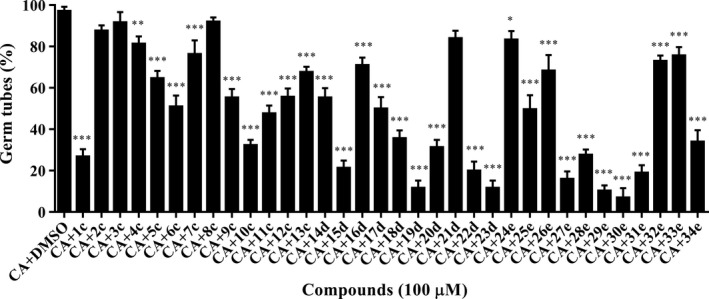
Effects of piperazine derivatives on *Candida albicans* hyphae formation. Each experiment was performed at least three times in triplicate, and each time at least 400 cells were counted for per treatment. CA:* C. albicans*. Compounds were dissolved in DMSO, and the same amount of DMSO as used as the solvent for compounds was used as control. Data are the mean ± standard deviation of three independent experiments. *P < 0.05; **P < 0.01; ***P < 0.001 (unpaired *t* test).

Yeast‐to‐hyphae transition is essential and required for *C. albicans* to infect human mucosal tissue (Lo *et al*., 1997; Saville *et al*., 2003; Sudbery *et al*., 2004; Finkel and Mitchell, 2011). The effects of piperazine derivatives on *C. albicans* morphological transition were then evaluated *in vitro* under hyphae induction conditions at 37°C. After induction for 4 h, the majority of *C. albicans* cells formed germ tubes in the control group, while hyphae formation was obviously inhibited with addition of many piperazine derivatives (Fig. 2). At least 16 compounds reduced hyphae formation in *C. albicans* cells by more than 50% when they were supplemented at a final concentration of 100 μM (Fig. 2).

### Piperazine derivatives inhibit *C. albicans* biofilm formation

**Figure 3:**
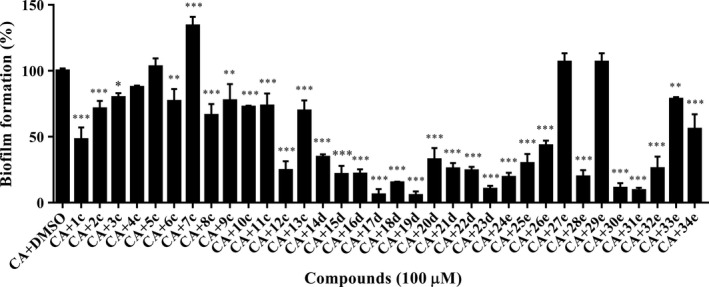
Effects of piperazine derivatives on *Candida albicans* biofilm formation. *C. albicans* cells were plated in the presence of compounds at a final concentration of 100 μM. CA:* C. albicans*. Compounds were dissolved in DMSO, and the same amount of DMSO as used as the solvent for compounds was used as control. *C. albicans* biofilm formed in the control group was arbitrarily defined as 100% and was used to normalize the biofilm of *C. albicans* formed with addition of compounds. Data are the mean ± standard deviation of three independent experiments. *P < 0.05; **P < 0.01; ***P < 0.001 (unpaired *t* test).

Biofilm formation by fungal pathogens on implanted medical devices is a serious medical problem (Ganguly and Mitchell, 2011). Cell adhesion and biofilm formation by *C. albicans* cells on host surfaces are also the first step in establishing infection. As piperazine derivatives showed excellent efficacy in preventing *C. albicans* hyphae formation, we then evaluated their inhibitory activity on *C. albicans* cell adhesion and biofilm formation on polystyrene. Among the 34 derivatives, 15 compounds strongly inhibited *C. albicans* biofilm formation by more than 70% (Fig. 3). Surprisingly, compounds **17d** and **19d** exhibited notably high suppression of *C. albicans* biofilm formation by more than 85% when they were supplemented at a final concentration of 100 μM (Fig. 3), suggesting piperazine derivatives are excellent anti‐biofilm agents against *C. albicans*. In addition, it was further indicated that different substitutions have various anti‐biofilm activities. Phenol derivatives (**1c‐13c**) exerted weak inhibitory activity, 2,4‐dichlorophenol derivatives (**14d‐23d**) showed strong inhibition, and 4‐hydroxybiphenyl derivatives (**24e‐34e**) exhibited moderate activity (Fig. 3), suggesting that the introduction of a halogen group improves the anti‐biofilm activity of piperazine compounds. Additionally, substitution of the benzene ring clearly influenced the activity of these compounds. Derivatives with 2,3‐CH_3_, 2,4‐CH_3_, 3,4‐Cl, 4‐OCH_2_CH_3_, 2‐OCH_3_ and 4‐Br (**19d**,** 23d**,** 24d**,** 28e**,** 30e**,** 31e**,** 32e**) exhibited strong inhibitory activity towards biofilm formation by *C. albicans* (Fig. 3, Table S1). In addition, the inhibitory activity of piperazine derivatives during hyphae formation correlated with their inhibition of biofilm formation by *C. albicans,* possibly indicating the vital role of hyphae formation in the *C. albicans* biofilm formation process.

### Piperazine derivatives attenuate *C. albicans* virulence

**Figure 4:**
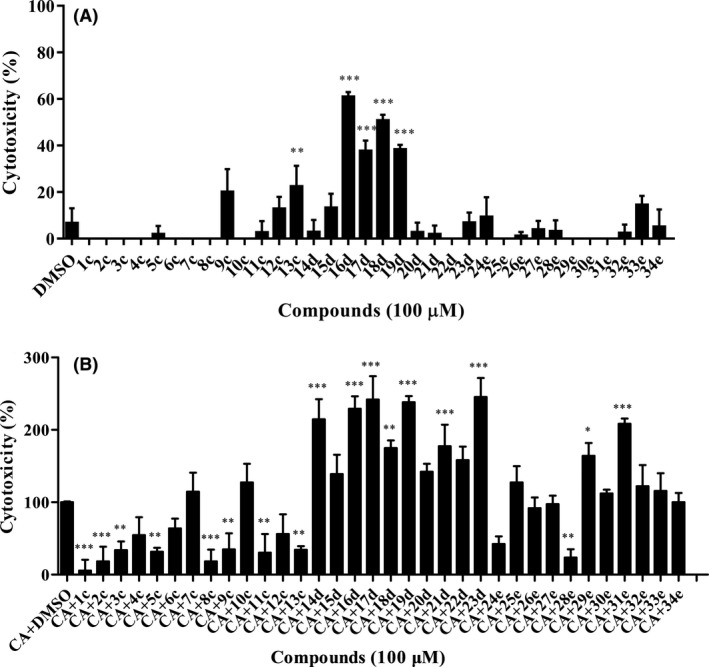
Effects of piperazine derivatives on *Candida albicans* virulence using a cell line. A. Analysis of the toxicity of compounds to A549 cells. Compounds were dissolved in DMSO, and the same amount of DMSO as used as the solvent for compounds was used as control. B. Analysis of the effect of compounds on the cytotoxicity of *C. albicans* to A549 cells. CA:* C. albicans*. Cell cytotoxicity was detected and measured as LDH release. The LDH released by A549 cells in (B) after inoculation with *C. albicans* in the absence of compounds was defined as 100% and used to normalize the LDH release ratios of other treatments. Data are the mean ± standard deviation of three independent experiments. *P < 0.05; **P < 0.01; ***P < 0.001 (unpaired *t* test).

To determine the effects of piperazine derivatives on the pathogenicity of *C. albicans*, we then investigated whether these compounds influenced *C. albicans* virulence in a cell line. Cytotoxicity was measured by quantifying the release of lactate dehydrogenase (LDH) into the supernatants of cultured A549 cells. It was found that most derivatives showed no toxic effects on A549 cells at a final concentration of 100 μM (Fig. 4A). However, the exogenous addition of certain piperazine derivatives led to a reduction in *C. albicans* cytotoxicity to A549 cells (Fig. 4B). Although 2,4‐dichlorophenol derivatives exhibited strong inhibition of biofilm formation, their toxicity to the cell line was also very high (Fig. 4A). Compounds **1c**,** 2c**,** 8c** and **28e** were highly effective at attenuating *C. albicans* cytotoxicity by more than 80% when they were supplemented at a final concentration of 100 μM, while they exerted no toxic effects on the cell line at 8‐h postinoculation (Fig. 4A and B). Considering that compounds **2c** and **8c** only weakly inhibited hyphae formation in *C. albicans*, compounds **1c** (1‐(4‐methoxyphenyl)‐4‐(3‐phenoxy‐2‐hydroxypropyl)‐piperazine) and **28e** (1‐(4‐ethoxyphenyl)‐4‐(1‐biphenylol‐2‐hydroxypropyl)‐piperazine) were then selected for further investigation. Interestingly, compounds **1c** and **28e** also inhibited the virulence of other clinical isolated *Candida* species (Fig. S1). However, some commercialized piperazine compounds showed no inhibition on *C. albicans* virulence even when they were added at a final concentration of 400 μM (Fig. S2).

### The inhibitory activity of piperazine derivatives on *C. albicans* hyphae formation, biofilm formation and virulence is dose dependent

**Figure 5:**
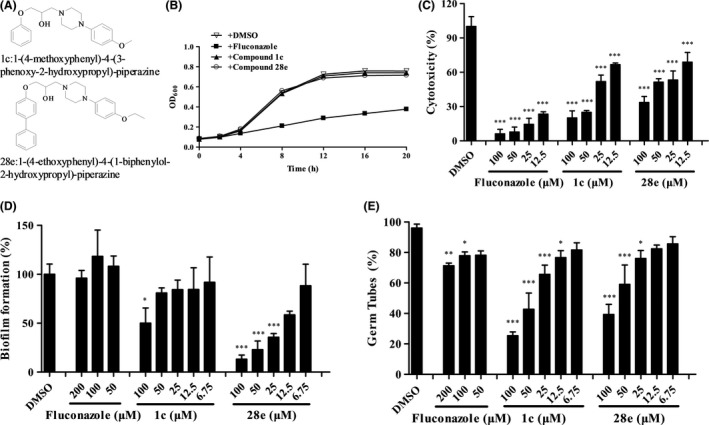
Effects of piperazine derivatives on *C. albicans* at different concentrations. (A) Structures of compounds **1c** and **28e**. (B) Effects of fluconazole and compounds **1c** and **28e** (100 μM) on the growth rate of *C. albicans* cells. The effects of different concentrations of fluconazole and compounds **1c** and **28e** on the virulence (C), biofilm formation (D) and hyphae formation (E) of *C. albicans*. Data are the mean ± standard deviation of three independent experiments. *P <* *0.05; **P <* *0.01; ***P <* *0.001 (unpaired *t* test).

To determine whether the effects of piperazine derivatives on *C. albicans* are related to their dosage, different concentrations of compounds **1c** and **28e** were assessed for their inhibitory activity during *C. albicans* morphological transition, biofilm formation and virulence (Fig. 5A). We also examined the effects of different concentrations of fluconazole, which is always used for clinical treatment. Both compounds **1c** and **28e** exhibited dose‐dependent activity in which they reduced *C. albicans* cytotoxicity by more than 50% at a final concentration of 50 μM but did not affect the growth rate of pathogenic cells (Fig. 5B and C). As a control, fluconazole severely inhibited the growth rate of *C. albicans* cells, suggesting that compounds **1c** and **28e** might be good candidates for development as novel anti‐virulence agents against *C. albicans* infections. The addition of compounds **1c** and **28e** at final concentrations of 6.75–100 μM decreased *C. albicans* biofilm formation by 10% to 50% and 13% to 85%, respectively, but the addition of fluconazole had no detectable effect (Fig. 5D). Additionally, compounds **1c** and **28e** inhibited hyphae formation by 20% to 78% and 16% to 65%, respectively (Fig. 5E), while most *C. albicans* cells formed short stick mycelia that were considered hyphae after the addition of fluconazole.

### Piperazine derivatives interfere with the morphological transition of *C. albicans* through multiple pathways

**Figure 6:**
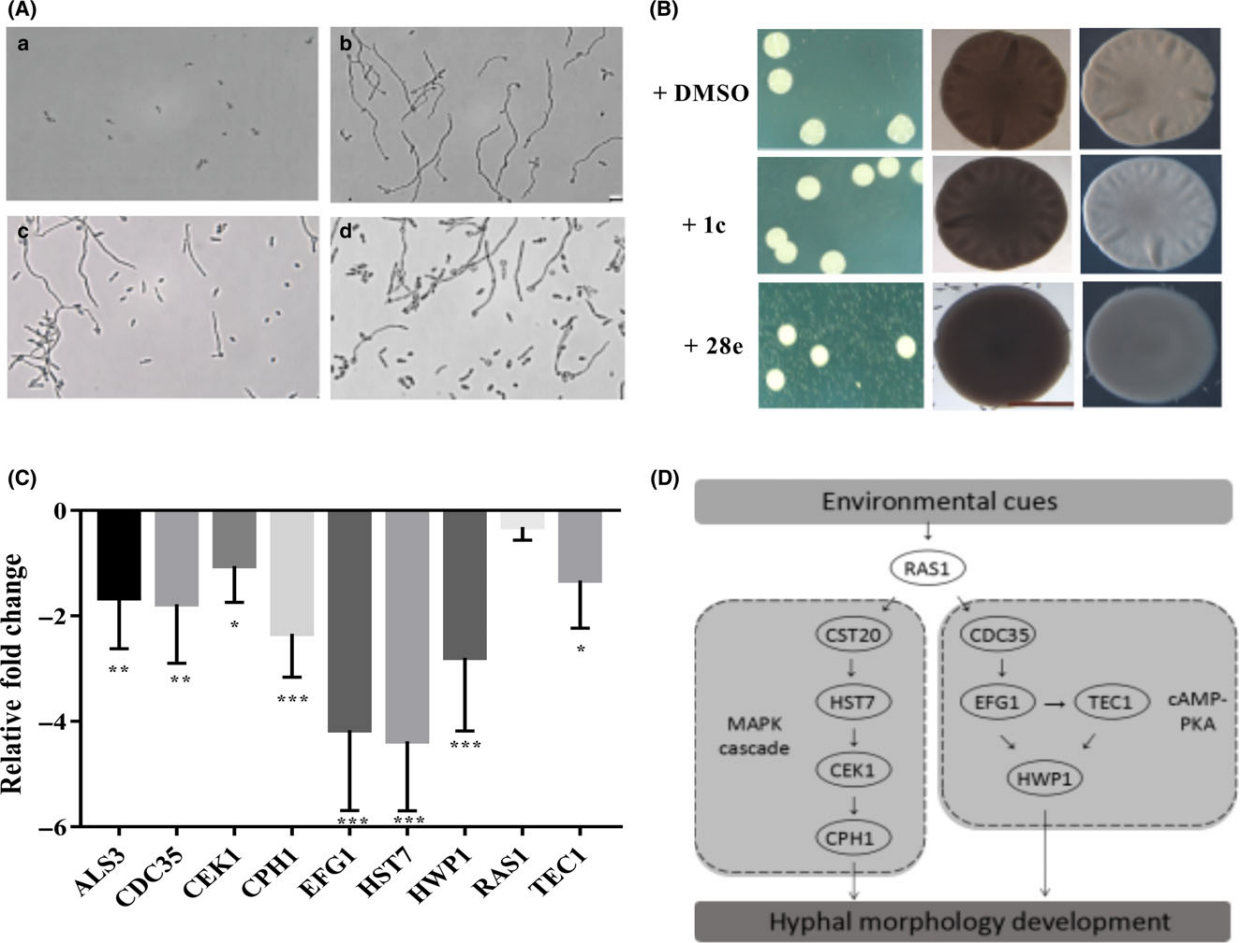
Influence of piperazine derivatives on *Candida albicans* morphology and hyphae formation. A. Effects of compounds **1c** and **28e** on hyphae formation of *C. albicans*. *C. albicans* cells were grown under non‐induction conditions (30°C) (a), or under induction conditions (37°C) (b). In (c and d), the cells were grown under the same condition as in (b) but treated with 100 μM of compound **1c** and compound **28e** respectively. The photographs were taken 4 h after induction. B. Effects of compounds **1c** and **28e** (100 μM) on colony morphology of *C. albicans*. C. Comparison of relative fold changes of regulator encoding genes between *C. albicans* cells with and without addition of compound **28e**. qRT‐PCR results were normalized using the *C*_*t*_ values obtained for the GSP1 amplifications run in the same plate. The relative levels of gene transcripts were determined from standard curves. Data are the mean ± standard deviation of three independent experiments. *P < 0.05; **P < 0.01; ***P < 0.001 (unpaired *t* test). D. Schematic diagram of the signalling pathways that govern hyphal morphogenesis in *C. albicans* affected by compound **28e**.

Besides the statistics of hyphae formation per cent, the photograph of hyphae formation was also provided to reflect the effect of the candidate compounds. After adding compounds **1c** and **28e**, the count of hyphae declined obviously (Fig. 6A). In good agreement with the inhibition of hyphae formation, compound **28e** also obviously affected the colony morphology of *C. albicans* (Fig. 6B). After addition of compound **28e**, the colonies were changed from wrinkled to slippery (Fig. 6B). To explore the working model of piperazine derivatives on the morphological transition of *C. albicans*, we continued to test whether these compounds interfered with the signalling pathways of the hyphae development process (Sudbery *et al*., 2004; Lu *et al*., 2011; Shareck and Belhumeur, 2011; Nobile *et al*., 2012). Real‐time PCR analysis showed that exogenous addition of the leading compound **28e** inhibited the expression levels of CDC35, EFG1, TEC1 and HWP1, which are regulators involved in the cAMP‐PKA pathways (Fig. 6C and D) (Sudbery, 2011). In addition, some regulators of the MAPK cascade (Zhao *et al*., 2013), such as HST7, CEK1 and CPH1, were also downregulated by the exogenous addition of compound **28e** (Fig. 6C and D). Collectively, these results demonstrated that compound **28e** influenced complex signal transduction pathways to interfere with the *C. albicans* filamentation process.

## Discussion

Our findings demonstrated for the first time that (1‐aryloxy‐2‐hydroxypropyl)‐phenylpiperazine compounds could be developed as potential antifungal agents to attenuate *C. albicans* virulence by targeting the filamentation process. The yeast‐to‐hyphae transition is always thought to be an indispensable step for invasion, and *C. albicans* mutants defective in morphological transitions are avirulent during infection (Braun *et al*., 2000; Saville *et al*., 2003; Zheng *et al*., 2003). The current clinical treatments against candidiasis caused by *C. albicans* almost always rely on limited antifungal agents, which are compromised by the limitation of drug development and the rapid emergence of drug‐resistant pathogens. Developing new strategies to treat fungal pathogens is a key research area for these reasons. As morphological transitions between yeast cells and filamentous forms play a vital role in pathogenesis, recent studies have focused on determining how to inhibit hyphae formation in fungal pathogens. Interestingly, some cell–cell communication signals, including farnesol, a *quorum sensing* signal produced by *C. albicans* and a BDSF *quorum sensing* signal from *Burkholderia cenocepacia*, show an excellent ability to inhibit *C. albicans* hyphae formation (Boon *et al*., 2008; Shirtliff *et al*., 2009). Our study here provided additional evidence that designing antifungal drugs using a function‐based approach that relies on the inhibition of hyphae formation but not the direct killing of pathogens is a feasible strategy.

Biofilms formed by *C. albicans* cells usually increase resistance to commonly used antifungal drugs, such as azoles and amphotericin B (Walker *et al*., 2010; Fiori *et al*., 2011). It is important to develop new antifungal drugs that efficiently control biofilm formation. Our results indicated that some piperazine derivatives showed a strong ability to inhibit *C. albicans* biofilm formation (Fig. 3). This ability was also consistent with their inhibition of hyphae formation (Fig. 2). As hyphae formation plays a vital role in *C. albicans* biofilm formation (Lu *et al*., 2014; Gulati and Nobile, 2016), the strategies to target morphological transition and biofilm development may be closely interlinked.

Piperazine is an important scaffold associated with several biological activities (Shaquiquzzaman *et al*., 2015; Singh *et al*., 2015). Many synthetic piperazine derivatives have been reported to exhibit promising antifungal activity by causing the death of fungal cells (Moraca *et al*., 2014; Thamban *et al*., 2017). However, our study verified for the first time that (1‐aryloxy‐2‐hydroxypropyl)‐phenylpiperazine scaffolds could be developed as antifungal agents against *C. albicans* using an anti‐virulence strategy. Another interesting characteristic of piperazine derivatives is that substitution on the benzene ring notably influences their activity (Figs 1, 2, 3, 4). We found that 2,4‐dichlorophenol derivatives showed strong anti‐biofilm activity but exhibited high toxicity against cell lines (Figs 3 and 4). Conversely, 4‐hydroxybiphenyl derivatives had high anti‐biofilm activity and low cytotoxicity, suggesting that they are appropriate candidates for further development. The novel compound **28e** containing 4‐hydroxybiphenyl and the 4‐OCH_2_CH_3_ moiety might be a promising potential agent against *C. albicans* and other clinical isolated Candida species given its excellent efficacy (Figs 5 and 6, S1). In general, our findings demonstrated that the high efficacy of (1‐aryloxy‐2‐hydroxypropyl)‐phenylpiperazine compounds in terms of both hyphae formation and biofilm formation by *C. albicans* increases their advantages for their development as new antifungal drugs.

## Experimental procedures

### Chemistry

^1^H NMR (500 MHz) spectra were recorded in CDCl_3_ or DMSO on a Bruker Avance spectrometer using tetramethylsilane (TMS) as an internal standard. Electrospray ionization (ESI) mass spectra (electron ionization (EI), 70 eV) were recorded on an Agilent 6330 ion trap LC/MS system. HRESI‐MS spectra were recorded using a Shimadzu LCMS‐IT‐TOF mass spectrometer. Thin‐layer chromatography (TLC) was performed on an aluminium plate precoated with silica gel and a fluorescence indicator (Merck, Darmstadt, Germany). The compounds were detected on the TLC plates using UV light (254 nm). All other reagents and chemicals were obtained from commercial sources and used as received unless otherwise stated.

### Strains, culture and agents

The *C. albicans* strain used in this study was the standard wild‐type strain SC5314 (ATCC^®^ MYA‐2876TM), which was grown either in 6.7 g l^−1^ yeast nitrogen broth without amino acids (YNB) with 2% glucose or in YPD medium (1% yeast extract, 2% peptone and 2% dextrose) at 30°C (non‐hyphae induction conditions) or 37°C (hyphae induction conditions). Human lung epithelial A549 cells were incubated in Dulbecco's modified Eagle's medium (DMEM) containing 10% fetal bovine serum (FBS).

### Biofilm formation assays

*Candida albicans* SC5314 strain was cultured overnight in YNB + 2% glucose media at 30°C and inoculated in the same medium to an OD_600_ of 0.5 in the presence of compounds at different concentrations, as indicated. One hundred microlitres of inoculated culture was grown in each well of a 96‐well polystyrene plate at 37°C for 4 h without shaking. The cultures were removed, and 0.02% crystal violet was added for 45 min. The plate was rinsed six to eight times with iced distilled water and decolorized with 200 μl of 75% ethanol. The quantity of crystal violet was determined by measuring the absorbance at 590 nm.

### Hyphal formation assays

An overnight culture of *C. albicans* SC5314 grown at 30°C was diluted in fresh YNB + 2% glucose to an OD_600_ of 0.1. Compounds were then added separately as indicated, and the cells were induced for 6 h at 37°C. Cells were centrifuged for 10 min at 5000 rpm, and the pellet was resuspended in 100 μl of fresh medium. Images of cells were captured using a Leica inverted fluorescence microscope with 20× magnification.

### Colony morphology

Spider medium agar plates (1% peptone, 1% mannitol, 0.2% K_2_HPO_4_ and 1.5% agar) were supplemented with different concentrations of compounds as indicated. *C. albicans* SC5314 cells were grown in the plates at 37°C for 24–30 h. Images of the colonies were obtained using a Leica DMi8 microscope and a Nikon Coolpix digital camera.

### Cell growth analysis

For the cell growth assay, *C. albicans* cells were cultured in YNB + 0.2% glucose and inoculated in the same medium to an OD_600_ of 0.05 in the absence or presence of compounds at final concentrations as indicated. Three hundred microlitres of inoculated culture was grown in each well at 30°C in a low‐intensity shaking model using a Bioscreen‐C Automated Growth Curves Analysis System (OY Growth Curves AB, Finland).

### Cytotoxicity assays

Cytotoxicity was assessed by measuring the release of LDH from A549 cells. The A549 cells were routinely grown in DMEM medium supplemented with 10% FBS in a 96‐well tissue culture plate with 1.5 × 10^4^ cells/well. Confluent A549 cells were washed and incubated with DMEM containing 1% FBS before infection. Overnight *C. albicans* cells were diluted to an OD_600_ of 0.1 with DMEM containing 1% FBS in the absence or presence of compounds at a final concentration as indicated. A549 cells were infected with fungal cells for 8 h. LDH in the supernatant was measured, and cytotoxicity was calculated relative to that of the uninfected control. Different strains and compounds were tested as the same way.

### Quantitative real‐time PCR

Real‐time PCR assays were conducted as described previously (Li *et al*., 2013, 2014). Overnight cultures of *C. albicans* cells grown in YNB + 0.2% glucose at 30°C were inoculated in the same medium to an OD_600_ of 0.1 in the absence or presence of compounds at a final concentration of 100 μM. After incubation for 6 h at 37°C, the cell samples were collected and washed with PBS. Total RNA was extracted using TRIzol (InVitrogen, Carlsbad, California, America) and quantified. cDNA was obtained through a reverse transcription reaction using a reverse transcription kit (TaKaRa Biotechnology) with the primers shown in Table S2, and real‐time PCR was performed with a 7300 plus real‐time PCR system (Applied Biosystems, America). The expression level of each gene was normalized to that of GSP1, which is a housekeeping gene in *C. albicans* cells (Clément *et al*., 2000). The relative expression levels of the target genes were calculated using the quantitation‐comparative CT(ΔΔ*C*_*t*_) method.

## Conflict of interests

The authors declare no conflict of interests.

## Supporting information


**Table S1.** Analysis of chemical structures of piperazine derivatives.
**Table S2.** PCR primers used in this study.
**Fig. S1.** Effects of piperazine derivatives on different clinic isolated *C. albicans* strains and other *Candida* species virulence using a cell line.
**Fig. S2.** Influences of different piperazine compounds on *C. albicans* SC5314 virulence using a cell line.
