# Supplementary material for: (1‐aryloxy‐2‐hydroxypropyl)‐phenylpiperazine derivatives suppress Candida albicans virulence by interfering with morphological transition
Source: Microb Biotechnol. 2018 Sep 17;11(6):1080–9. doi: 10.1111/1751-7915.13307 (PMC6196381; doi:10.1111/1751-7915.13307)
Supplement: Supplementary file 1 — Table S1. Analysis of chemical structures of piperazine derivatives. Table S2. PCR primers used in this study. Fig. S1. Effects of piperazine derivatives on different clinic isolated C. albicans strains and other Candida species virulence using a cell line. Fig. S2. Influences of different piperazine compounds on C. albicans SC5314 virulence using a cell line. [file MBT2-11-1080-s001.pdf]

Supplementary Information:

Zhao et al.,

(1-aryloxy-2-hydroxypropyl)-phenylpiperazine derivatives suppress *Candida albicans* virulence by interfering with morphological transition

Table S1. Analysis of chemical structures of piperazine derivatives

Com. No.	Structure	Description of Mass Spectra	Description of NMR Spectra
**1c**	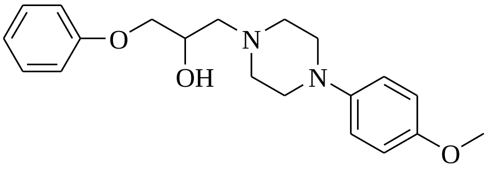	ESI (*m/z*): $[M+H]^+$ 343.3.<br><br>HRESIMS *m/z*: 343.2026 (M^++H, calcd for $C_{20}H_{27}N_2O_3$, 343.1943).	^1H NMR (500 MHz, DMSO), δ (ppm): 6.79-7.29 (m, 9H, ArH), 3.96-3.98 (m, 2H, -OCH_2 -), 3.85-3.88 (m, 1H, -CH-OH), 3.66 (s, 3H, -OCH_3-), 3.50 (brs, 2H, -CH_2-N), 2.97-2.99 (brs, 4H, piperazinyl right H), 2.54-2.62 (m, 4H, piperazinyl left H).
**2c***	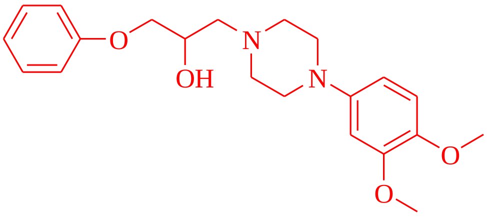	ESI (*m/z*): $[M+H]^+$ 373.3.<br><br>HRESIMS *m/z*: 373.2123 (M^++H, calcd for $C_{21}H_{29}N_2O_4$, 373.2049).	^1H NMR (500 MHz, DMSO), δ (ppm): 6.91-7.40 (m, 9H, ArH), 3.99-4.01 (m, 2H, -OCH_2 -), 3.87-3.91 (m, 1H, -CH-OH), 3.57 (s, 2H, -CH_2-N), 2.97 (brs, 4H, piperazinyl right H), 2.63 (m, 4H, piperazinyl left H), 2.50-2.55 (m, 2H, -CH_2-N).
**3c**	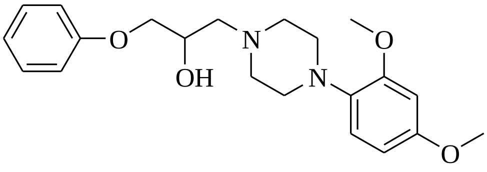	ESI (*m/z*): $[M+H]^+$ 373.3.<br><br>HRESIMS *m/z*: 373.2132 (M^++H, calcd for $C_{21}H_{28}ClN_2O_4$, 373.2049).	^1H NMR (500 MHz, DMSO), δ (ppm): 6.40-7.29 (m, 8H, ArH), 3.96-3.98 (m, 2H, -OCH_2 -), 3.83-3.87 (m, 1H, -CH-OH), 3.72 (s, 3H, -OCH_3), 3.68 (s, 3H, -OCH_3), 3.57 (s, 2H, -CH_2-N), 2.84 (brs, 4H, piperazinyl right H), 2.55 (m, 4H, piperazinyl left H).
**4c***	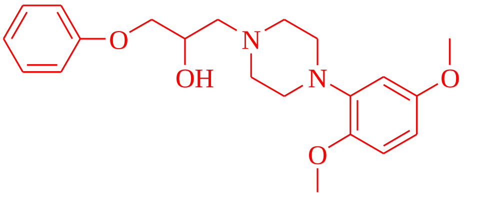	ESI (*m/z*): $[M+H]^+$ 373.3.<br><br>HRESIMS *m/z*: 373.2132 (M^++H, calcd for $C_{21}H_{29}N_2O_4$, 373.2049).	^1H NMR (500 MHz, DMSO), δ (ppm): 6.41-7.28 (m, 8H, ArH), 3.96-3.98 (m, 2H, -OCH_2 -), 3.84-3.88 (m, 1H, -CH-OH), 3.69 (s, 3H, -OCH_3), 3.65 (s, 3H, -OCH_3), 2.94 (brs, 4H, piperazinyl right H), 2.57 (m, 4H, piperazinyl left H), 2.47-2.51 (m, 2H, -CH_2-N).
**5c***	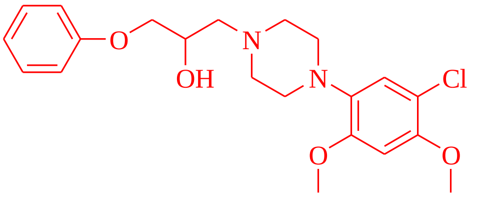	ESI (*m/z*): $[M+H]^+$ 407.3.<br><br>HRESIMS *m/z*: 407.1738 (M^++H, calcd for $C_{21}H_{28}ClN_2O_4$, 407.1659).	^1H NMR (500 MHz, DMSO), δ (ppm): 6.76-7.30 (m, 7H, ArH), 3.98-4.00 (m, 2H, -OCH_2 -), 3.85-3.89 (m, 1H, -CH-OH), 3.82 (s, 6H, -OCH_3×2), 2.87 (brs, 4H, piperazinyl right H), 2.56-2.59 (brs, 4H, piperazinyl left H), 2.48-2.52 (brs, 2H, -CH_2-N).

**6c***	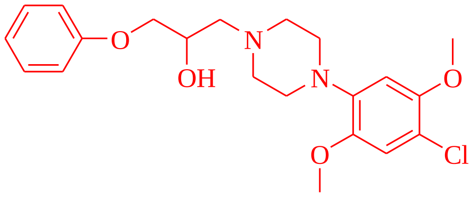	ESI (*m/z*): [M+H]+ 407.3.<br><br>HRESIMS *m/z*: 407.1742 (M^{+}+H, calcd for $C_{21}H_{28}ClN_2O_4$, 407.1659).	^{1}H NMR (500 MHz, DMSO), δ (ppm): 6.63-7.30 (m, 7H, ArH), 3.97-4.01 (m, 2H, -OCH_2 -), 3.86-3.88 (m, 1H, -C<u>H</u>-OH), 3.79 (s, 3H, -OCH_3), 3.74 (s, 3H, -OCH_3), 3.00 (brs, 4H, piperazinyl right H), 2.59 (brs, 4H, piperazinyl left H), 2.50-2.52 (m, 2H, -CH_2-N).
**7c***	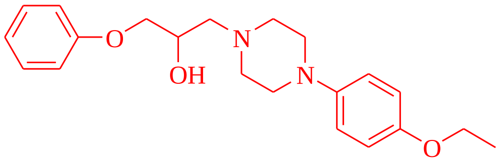	ESI (*m/z*): [M+H]+ 357.3.<br><br>HRESIMS *m/z*: 357.2178 (M^{+}+H, calcd for $C_{21}H_{29}N_2O_3$, 357.21).	^{1}H NMR (500 MHz, DMSO), δ (ppm): 6.83-7.28 (m, 9H, ArH), 3.96-3.98 (m, 4H, -OCH_2-×2), 3.86 (brs, 1H, -C<u>H</u>-OH), 2.95 (brs, 4H, piperazinyl right H), 2.58 (brs, 4H, piperazinyl left H), 2.43 (m, 2H, -CH_2-N), 1.31 (t, 3H, -CH_3).
**8c***	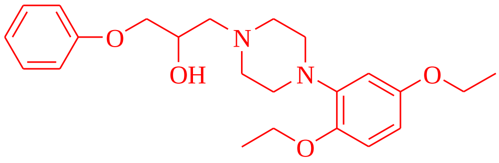	ESI (*m/z*): [M+H]+ 401.3.<br><br>HRESIMS *m/z*: 401.2440 (M^{+}+H, calcd for $C_{23}H_{33}N_2O_4$, 401.2362).	^{1}H NMR (500 MHz, DMSO), δ (ppm): 6.39-7.30 (m, 8H, ArH), 3.97-4.00 (m, 2H, -OCH_2 -), 3.90-3.95 (m, 4H, -OC<u>H</u>$_2CH_3$×2), 3.87-3.88 (m, 1H, -C<u>H</u>-OH), 2.98 (brs, 4H, piperazinyl right H), 2.59 (brs, 4H, piperazinyl left H), 2.52 (m, 2H, -CH_2-N), 1.27-1.31 (m, 6H, -CH_3×2).
**9c**	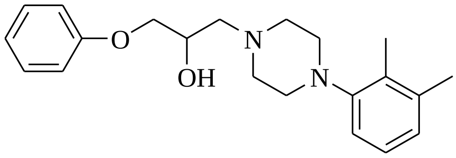	ESI (*m/z*): [M+H]+ 341.3.<br><br>HRESIMS *m/z*: 341.2233 (M^{+}+H, calcd for $C_{21}H_{29}N_2O_2$, 341.2151).	^{1}H NMR (500 MHz, DMSO), δ (ppm): 6.82-7.28 (m, 8H, ArH), 3.96-4.00 (m, 2H, -OCH_2 -), 3.85-3.88 (m, 1H, -C<u>H</u>-OH), 2.76 (brs, 4H, piperazinyl right H), 2.59 (brs, 4H, piperazinyl left H) 2.42-2.53 (m, 2H, -CH_2-N), 2.17 (s, 3H,-CH_3), 2.12 (s, 3H,-CH_3).
**10c***	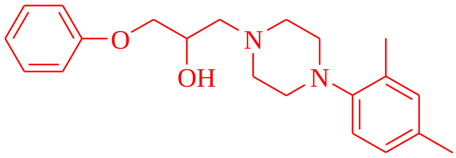	ESI (*m/z*): [M+H]+ 341.3.<br><br>HRESIMS *m/z*: 341.2233 (M^{+}+H, calcd for $C_{21}H_{29}N_2O_2$, 341.2151).	^{1}H NMR (500 MHz, DMSO), δ (ppm): 6.88-7.30 (m, 8H, ArH), 3.99-4.01 (m, 2H, -OCH_2 -), 3.86-3.90 (m, 1H, -C<u>H</u>-OH), 2.78 (brs, 4H, piperazinyl right H), 2.59 (brs, 4H, piperazinyl left H) 2.50-2.54 (m, 2H, -CH_2-N), 2.19 (s, 6H,-CH_3×2).
**11c**	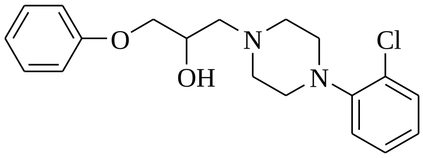	ESI (*m/z*): [M+H]+ 347.2.<br><br>HRESIMS *m/z*: 347.1533 (M^{+}+H, calcd for $C_{19}H_{24}ClN_2O_2$,	^{1}H NMR (500 MHz, DMSO), δ (ppm): 6.91-7.40 (m, 9H, ArH), 3.99-4.01 (m, 2H, -OCH_2 -), 3.87-3.91 (m, 1H, -C<u>H</u>-OH), 3.57 (s, 2H, -CH_2-N), 2.97 (brs, 4H, piperazinyl right H), 2.63 (m, 4H, piperazinyl left H), 2.50-2.55

		347.1448).	(m, 2H, -CH_2-N).
**12c**	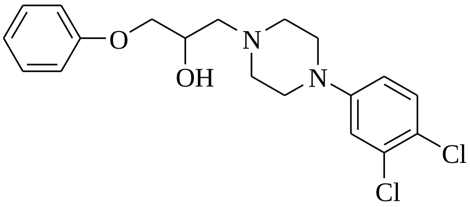	ESI (*m/z*): $[M+H]^+$ 381.3. HRESIMS *m/z*: 381.1139 (M^++H, calcd for $C_{19}H_{23}Cl_2N_2O_2$, 381.1058).	^1H NMR (500 MHz, DMSO), δ (ppm): 6.86-7.35 (m, 8H, ArH), 3.94-3.99 (m, 2H, -OCH_2 -), 3.84-3.88 (m, 1H, -CH-OH), 3.12 (brs, 4H, piperazinyl right H), 2.53-2.58 (m, 4H, piperazinyl left H), 2.47-2.52 (m, 2H, -CH_2-N).
**13c***	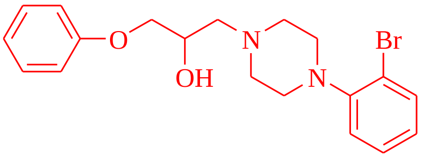	ESI (*m/z*): $[M+H]^+$ 391.2. HRESIMS *m/z*: 391.1021 (M^++H, calcd for $C_{19}H_{24}BrN_2O_2$, 391.0943).	^1H NMR (500 MHz, DMSO), δ (ppm): 6.92-7.55 (m, 9H, ArH), 3.97-3.99 (m, 2H, -OCH_2 -), 3.87 (brs, 1H, -CH-OH), 2.93 (brs, 4H, piperazinyl right H), 2.61 (brs, 4H, piperazinyl left H), 2.51 (m, 2H, -CH_2-N).
**14d**	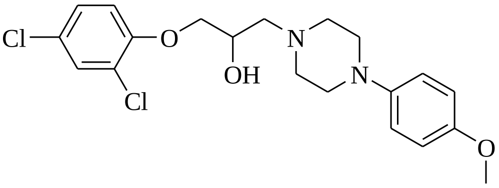	ESI (*m/z*): $[M+H]^+$ 411.2. HRESIMS *m/z*: 411.1244 (M^++H, calcd for $C_{20}H_{25}Cl_2N_2O_3$, 411.1164).	^1H NMR (500 MHz, DMSO), δ (ppm): 6.77-7.51 (m, 7H, ArH), 4.03-4.06 (m, 1H, -CH-OH), 3.97-4.02 (m, 2H, -OCH_2 -), 3.65 (s, 3H, -OCH_3), 2.95 (brs, 4H, piperazinyl right H), 2.52-2.57 (m, 4H, piperazinyl left H), 2.50 (brs, 2H, -CH_2-N).
**15d***	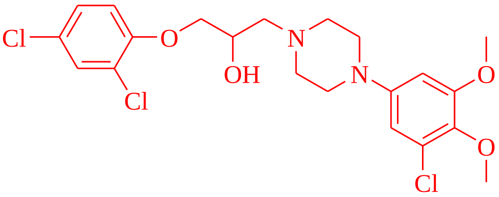	ESI (*m/z*): $[M+H]^+$ 475.3. HRESIMS *m/z*: 475.0962 (M^++H, calcd for $C_{21}H_{26}Cl_3N_2O_4$, 475.088).	^1H NMR (500 MHz, DMSO), δ (ppm): 6.71-7.48 (m, 5H, ArH), 4.03-4.05 (m, 1H, -CH-OH), 3.96-3.98 (m, 2H, -OCH_2 -), 3.79 (s, 6H, -OCH_3×2), 2.83 (brs, 4H, piperazinyl right H), 2.54 (m, 4H, piperazinyl left H), 2.50 (brs, 2H, -CH_2-N).
**16d***	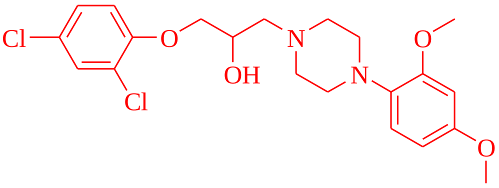	ESI (*m/z*): $[M+H]^+$ 441.3. HRESIMS *m/z*: 441.1350 (M^++H, calcd for $C_{21}H_{27}Cl_2N_2O_4$, 441.127).	^1H NMR (500 MHz, DMSO), δ (ppm): 6.38-7.49 (m, 6H, ArH), 4.04-4.05 (m, 1H, -CH-OH), 3.98 (brs, 2H, -OCH_2 -), 3.72 (s, 3H, -OCH_3), 3.67 (s, 3H, -OCH_3), 2.82 (brs, 4H, piperazinyl right H), 2.54 (m, 4H, piperazinyl left H), 2.50 (brs, 2H, -CH_2-N).
**17d***	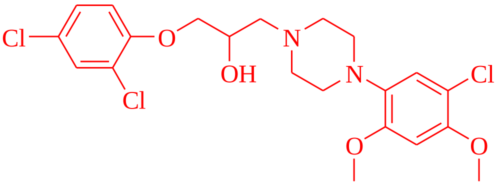	ESI (*m/z*): $[M+H]^+$ 475.3. HRESIMS *m/z*: 475.0959 (M^+ + H, calcd for $C_{21}H_{26}Cl_3N_2O_4$, 475.088).	^1H NMR (500 MHz, DMSO), δ (ppm): 6.60-7.52 (m, 5H, ArH), 4.05-4.07 (m, 1H, -CH-OH), 3.98-4.00 (m, 2H, -OCH_2 -), 3.77 (s, 3H, -OCH_3), 3.72 (s, 3H, -OCH_3), 2.97 (brs, 4H, piperazinyl right H), 2.56 (brs, 4H, piperazinyl left H), 2.50 (brs,

			2H, $-CH_2$-N).
**18d***	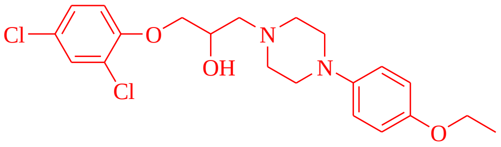	ESI (*m/z*): [M+H]$^+$ 425.3.<br><br>HRESIMS *m/z*: 425.1393 (M^++H, calcd for $C_{21}H_{27}Cl_2N_2O_3$, 425.132).	^{1}H NMR (500 MHz, DMSO), δ (ppm): 6.82-7.51 (m, 7H, ArH), 3.94-3.99 (m, 4H, $-OCH_2$-×2), 4.04-4.06 (brs, 1H, -CH-OH), 2.94 (brs, 4H, piperazinyl right H), 2.57 (brs, 4H, piperazinyl left H), 2.43 (m, 2H, $-CH_2$-N), 1.30 (t, 3H, $-CH_3$).
**19d***	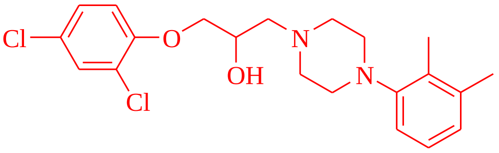	ESI (*m/z*): [M+H]$^+$ 409.3.<br><br>HRESIMS *m/z*: 409.1442 (M^++H, calcd for $C_{21}H_{27}Cl_2N_2O_2$, 409.1371).	^{1}H NMR (500 MHz, DMSO), δ (ppm): 6.83-7.53 (m, 6H, ArH), 4.05-4.07 (m, 1H, -CH-OH), 3.99-4.00 (m, 2H, $-OCH_2$ -), 2.75 (brs, 4H, piperazinyl right H), 2.56 (brs, 4H, piperazinyl left H), 2.46 (brs, 2H, $-CH_2$-N), 2.17 (s, 3H, $-CH_3$), 2.12 (s, 3H, $-CH_3$).
**20d***	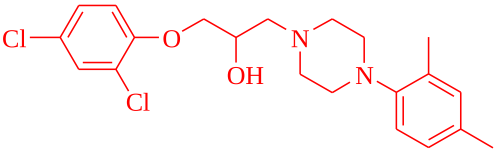	ESI (*m/z*): [M+H]$^+$ 409.3.<br><br>HRESIMS *m/z*: 409.1453 (M^++H, calcd for $C_{21}H_{27}Cl_2N_2O_2$, 409.1371).	^{1}H NMR (500 MHz, DMSO), δ (ppm): 6.83-7.50 (m, 6H, ArH), 4.04-4.06 (m, 1H, -CH-OH), 3.97-4.01 (m, 2H, $-OCH_2$ -), 2.73 (brs, 4H, piperazinyl right H), 2.55 (brs, 4H, piperazinyl left H), 2.44 (brs, 2H, $-CH_2$-N), 2.16 (s, 3H, $-CH_3$), 2.15 (s, 3H, $-CH_3$).
**21d***	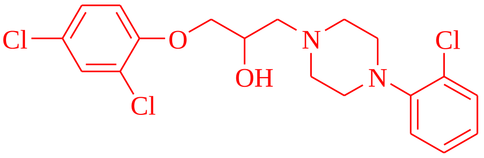	ESI (*m/z*): [M+H]$^+$ 415.2.<br><br>HRESIMS *m/z*: 415.0752 (M^++H, calcd for $C_{19}H_{22}Cl_3N_2O_2$, 415.0669).	^{1}H NMR (500 MHz, DMSO), δ (ppm): 7.00-7.53 (m, 7H, ArH), 4.05-4.07 (m, 1H, -CH-OH), 3.99 (brs, 2H, $-OCH_2$ -), 2.94 (brs, 4H, piperazinyl right H), 2.61 (brs, 4H, piperazinyl left H), 2.55 (brs, 2H, $-CH_2$-N).
**22d***	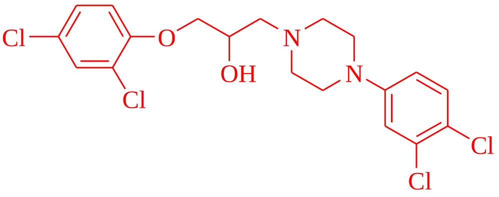	ESI (*m/z*): [M+H]$^+$ 449.1.<br><br>HRESIMS *m/z*: 449.0364 (M^+ + H, calcd for $C_{19}H_{21}Cl_4N_2O_2$, 449.0279).	^{1}H NMR (500 MHz, DMSO), δ (ppm): 6.83-7.47 (m, 6H, ArH), 4.03-4.04 (m, 1H, -CH-OH), 3.97-4.02 (m, 2H, $-OCH_2$ -), 3.10 (brs, 4H, piperazinyl right H), 2.54 (brs, 4H, piperazinyl left H), 2.44 (brs, 2H, $-CH_2$-N).
**23d***	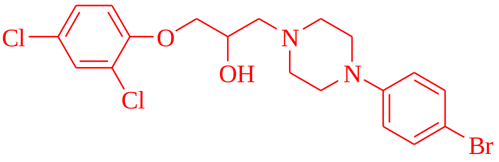	ESI (*m/z*): [M+H]$^+$ 461.1.<br><br>HRESIMS *m/z*: 461.0223 (M^++H, calcd for $C_{19}H_{22}BrCl_2N_2O_2$, 461.1922).	^{1}H NMR (500 MHz, DMSO), δ (ppm): 6.81-7.49 (m, 7H, ArH), 4.02-4.05 (m, 2H, $-OCH_2$ -), 3.96-4.01 (m, 1H, -CH-OH), 3.05-3.06 (brs, 4H, piperazinyl right H), 2.55-2.56 (m, 4H, piperazinyl left H), 2.50 (brs, 2H, $-CH_2$-N).

**24e**	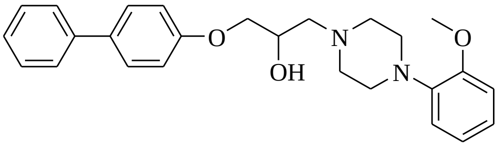	ESI (*m/z*): $[M+H]^+$ 419.3.<br><br>HRESIMS *m/z*: 419.2340 (M^++H, calcd for $C_{26}H_{31}N_2O_3$, 419.2256).	^{1}H NMR (500 MHz, DMSO), δ (ppm): 6.85-7.59 (m, 13H, ArH), 4.00-4.04 (m, 4H, -OCH_2-), 3.90-3.93 (m, 1H, -CH-OH), 3.74 (s, 3H, -OCH_3), 2.94 (brs, 4H, piperazinyl right H), 2.59 (brs, 4H, piperazinyl left H), 2.44 (m, 2H, -CH_2-N).
**25e**	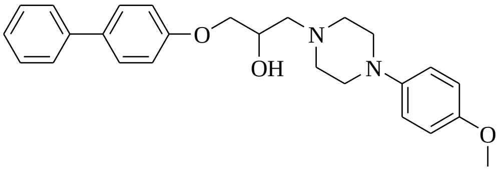	ESI (*m/z*): $[M+H]^+$ 419.3.<br><br>HRESIMS *m/z*: 419.2235 (M^++H, calcd for $C_{26}H_{31}N_2O_3$, 419.2256).	^{1}H NMR (500 MHz, DMSO), δ (ppm): 6.80-7.62 (m, 13H, ArH), 4.00-4.06(m, 2H, -OCH_2 -), 3.92-3.95 (m, 1H, -CH-OH), 3.67 (s, 3H, -OCH_3), 3.00-3.02 (m, 4H, piperazinyl right H), 2.56-2.65 (brs, 4H, piperazinyl left H), 2.41-2.45 (m, 2H, -CH_2-N).
**26e***	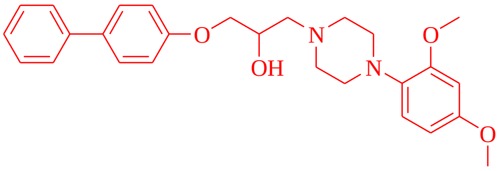	ESI (*m/z*): $[M+H]^+$ 449.3.<br><br>HRESIMS *m/z*: 449.2440 (M^++H, calcd for $C_{27}H_{33}N_2O_4$, 449.2362).	^{1}H NMR (500 MHz, DMSO), δ (ppm): 6.39-7.59 (m, 12H, ArH), 4.00-4.03 (m, 2H, -OCH_2 -), 3.89-3.92 (m, 1H, -CH-OH), 3.72 (s, 3H, -OCH_3), 3.67 (s, 3H, -OCH_3), 3.61 (s, 2H, -CH_2-N), 2.85 (brs, 4H, piperazinyl right H), 2.56 (brs, 4H, piperazinyl left H).
**27e***	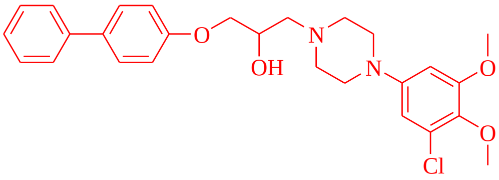	ESI (*m/z*): $[M+H]^+$ 483.3.<br><br>HRESIMS *m/z*: 483.2049 (M^++H, calcd for $C_{27}H_{32}BrN_2O_4Cl$, 483.1972).	^{1}H NMR (500 MHz, DMSO), δ (ppm): 6.73-7.59 (m, 11H, ArH), 3.99-4.02 (m, 2H, -OCH_2-), 3.89-3.92 (m, 1H, -CH-OH), 3.80 (s, 6H, -OCH_3×2), 2.85 (brs, 4H, piperazinyl right H), 2.56 (brs, 4H, piperazinyl left H), 2.44 (brs, 2H, -CH_2-N).
**28e***	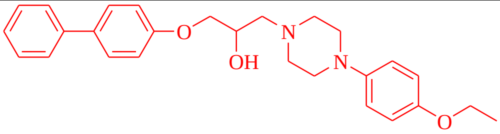	ESI (*m/z*): $[M+H]^+$ 433.3.<br><br>HRESIMS *m/z*: 433.2497 (M^++H, calcd for $C_{27}H_{33}N_2O_3$, 433.2413).	^{1}H NMR (500 MHz, DMSO), δ (ppm): 6.84-7.59 (m, 13H, ArH), 3.94-4.03 (m, 4H, -OCH_2-×2), 3.91-3.93 (m, 1H, -CH-OH), 2.96 (brs, 4H, piperazinyl right H), 2.59 (brs, 4H, piperazinyl left H), 2.44 (m, 2H, -CH_2-N), 1.31 (t, 3H, -CH_3).
**29e***	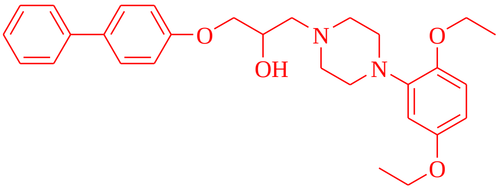	ESI (*m/z*): $[M+H]^+$ 477.4.<br><br>HRESIMS *m/z*: 477.2755 (M^++H, calcd for $C_{29}H_{37}N_2O_4$, 477.2675).	^{1}H NMR (500 MHz, DMSO), δ (ppm): 6.37-7.58 (m, 12H, ArH), 4.01-4.03 (m, 2H, -OCH_2 -), 3.85-3.92 (m, 4H, -OCH_2CH_3×2), 3.64 (s, 2H, -CH_2-N), 2.96 (brs, 4H, piperazinyl right H), 2.57 (brs, 4H, piperazinyl left H), 2.40-2.43 (m, 1H, -CH-OH),

			1.24-1.27 (m, -6H, $-CH_3 \times 2$).
**30e**	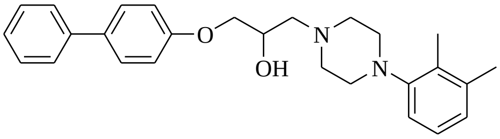	ESI (*m/z*): $[M+H]^+$ 417.3.<br><br>HRESIMS *m/z*: 417.2543 (M^++H, calcd for $C_{27}H_{33}N_2O_2$, 417.2464).	^1H NMR (500 MHz, DMSO), δ (ppm): 6.88-7.61 (m, 12H, ArH), 4.01-4.07 (m, 2H, $-OCH_2$ -), 3.92-3.95 (m, 1H, -CH-OH), 2.79 (brs, 4H, piperazinyl right H), 2.60 (brs, 4H, piperazinyl left H), 2.43-2.52 (m, 2H, $-CH_2$-N), 2.19 (s, 6H, $-CH_3 \times 2$).
**31e***	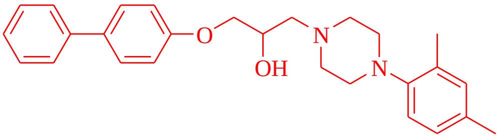	ESI (*m/z*): $[M+H]^+$ 417.3.<br><br>HRESIMS *m/z*: 417.2546 (M^++H, calcd for $C_{27}H_{33}N_2O_2$, 417.2464).	^1H NMR (500 MHz, DMSO), δ (ppm): 6.82-7.59 (m, 12H, ArH), 4.01-4.04 (m, 2H, $-OCH_2$ -), 3.90-3.93 (m, 1H, -CH-OH), 2.76 (brs, 4H, piperazinyl right H), 2.60 (brs, 4H, piperazinyl left H), 2.43-2.50 (m, 2H, $-CH_2$-N), 2.17 (s, 3H, $-CH_3$), 2.11 (s, 3H, $-CH_3$).
**32e***	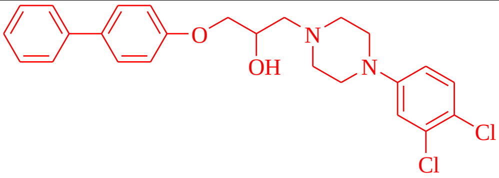	ESI (*m/z*): $[M+H]^+$ 457.3.<br><br>HRESIMS *m/z*: 457.1451 (M^++H, calcd for $C_{25}H_{27}Cl_2N_2O_2$, 457.1371).	^1H NMR (500 MHz, DMSO), δ (ppm): 6.89-7.60 (m, 12H, ArH), 4.01-4.04 (m, 2H, $-OCH_2$ -), 3.90-3.93 (m, 1H, -CH-OH), 3.15 (brs, 4H, piperazinyl right H), 2.57 (m, 4H, piperazinyl left H), 2.40-2.52 (m, 2H, $-CH_2$-N).
**33e***	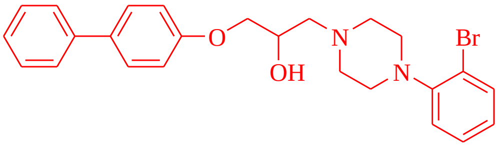	ESI (*m/z*): $[M+H]^+$ 467.2.<br><br>HRESIMS *m/z*: 467.1334 (M^++H, calcd for $C_{25}H_{28}BrN_2O_2$, 467.1256).	^1H NMR (500 MHz, DMSO), δ (ppm): 6.94-7.60 (m, 13H, ArH), 4.00-4.05 (m, 2H, $-OCH_2$ -), 3.91-3.93 (m, 1H, -CH-OH), 2.95 (brs, 4H, piperazinyl right H), 2.63 (brs, 4H, piperazinyl left H), 2.43-2.52 (m, 2H, $-CH_2$-N).
**34e***	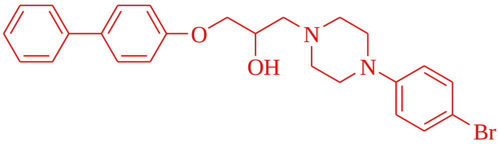	ESI (*m/z*): $[M+H]^+$ 467.1.<br><br>HRESIMS *m/z*: 467.1334 (M^++H, calcd for $C_{25}H_{28}BrN_2O_2$, 467.1256).	^1H NMR (500 MHz, $CDCl_3$), δ (ppm): 6.79-7.56 (m, 13H, ArH), 4.15-4.19 (m, 1H, -CH-OH), 4.06-4.07 (m, 2H, $-OCH_2$-), 3.18-3.21 (m, 4H, piperazinyl right H), 2.83-2.87 (m, 2H, $-CH_2$-N), 2.60-2.70 (m, 4H, piperazinyl left H).

Note: Structures with * are novel compounds.

Table S2. PCR primers used in this study

Primer	Sequence (5'-3')
GSP1-F[a]	TGAAGTCCATCCATTAGGAT
GSP1-R	ATCTCTATGCCAGTTTGGAA
RAS1-F	GGCCATGAGAGAACAATATA
RAS1-R	GTCTTTCCATTTCTAAATCAC
CDC35-F	TTCATCAGGGGTTATTTCAC
CDC35-R	CTCTATCAACCCGCCATTTC
PDE2-F	ACCACCACCACTACTACTAC
PDE2-R	AAAATGAGTTGTTCCTGTCC
EFG1-F	TATGCCCCAGCAAACAACTG
EFG1-R	TTGTTGTCCTGCTGTCTGTC
TEC1-F	AGGTTCCCTGGTTTAAGTG
TEC1-R	ACTGGTATGTGTGGGTGAT
CST20-F	TTCTGACTTCAAAGACATCAT
CST20-R	AATGTATATTTCTGGTGGTG
HST7-F	ACTCCAACATCCAATATAACA
HST7-R	TTGATTGACGTTCAATGAAGA
CEK1-F	AGCTATACAACGACCAATTAA
CEK1-R	CATTAGCTGAATGCATAGCT
CPH1-F	ATGCAACACTATTTATACCTC
CPH1-R	CGGATATTGTTGATGATGATA
ALS3-F	CTAATGCTGCTACGTATAATT

ALS3-R	CCTGAAATTGACATGTAGCA
HWP1-F	TGGTGCTATTACTATTCCGG
HWP1-R	CAATAATAGCAGCACCGAAG
ECE1-F	GCTGGTATCATTGCTGATAT
ECE1-R	TTCGATGGATTGTTGAACAC

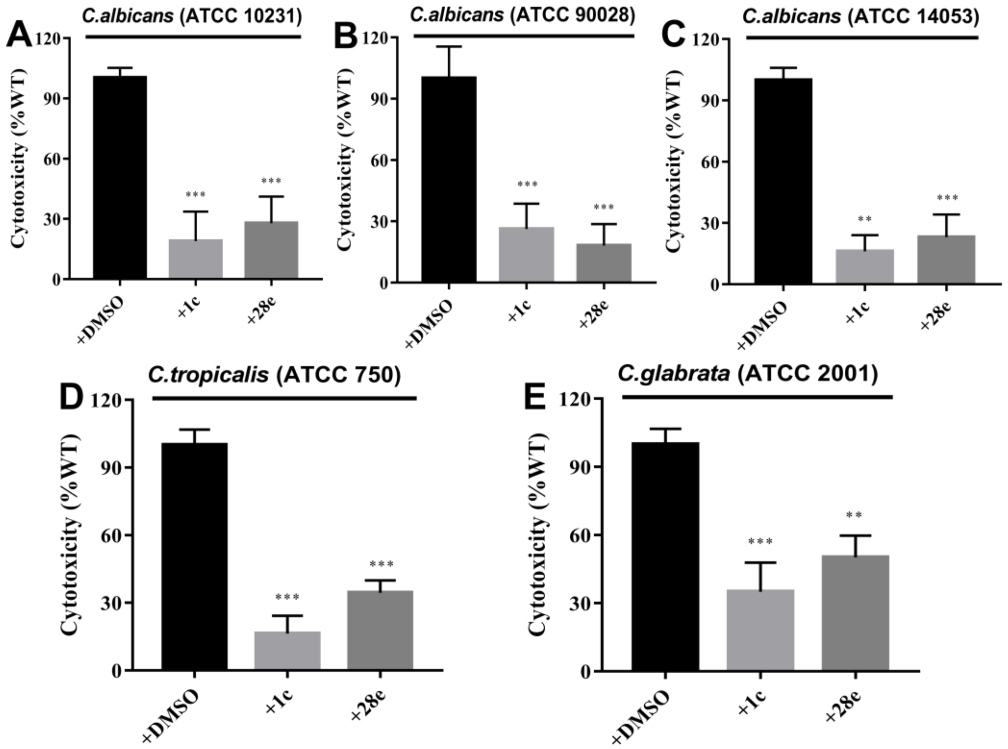

Fig. S1. Effects of piperazine derivatives on different clinic isolated *C. albicans* strains and other *Candida* species virulence using a cell line. Cytotoxicity assays were performed with the same methods as used in Fig 4B. The final concentration of compound 1c and 28e was 100 μM for the treatment of five strains. Data are the mean ± standard deviation of three independent experiments. *, $P < 0.05$; **, $P < 0.01$; ***, $P < 0.001$ (unpaired *t* test).

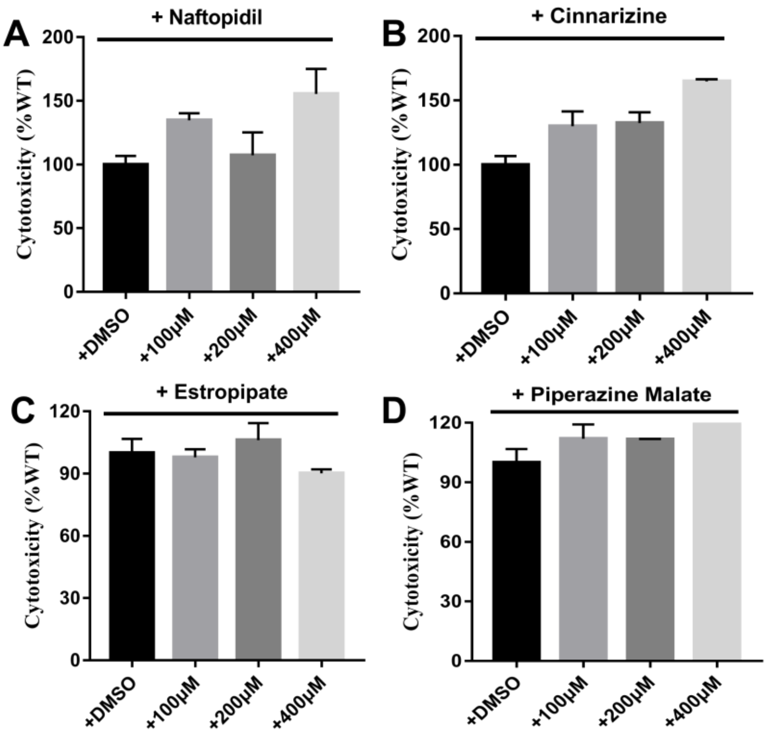

Fig. S2. Influences of different piperazine compounds on *C. albicans* SC5314 virulence using a cell line. Data are the mean ± standard deviation of three independent experiments.
